# One-Year Quality of Life Trends in Early-Stage Lung Cancer Patients After Lobectomy

**DOI:** 10.3389/fpsyg.2020.534428

**Published:** 2020-12-10

**Authors:** Chiara Marzorati, Ketti Mazzocco, Dario Monzani, Francesca Pavan, Monica Casiraghi, Lorenzo Spaggiari, Massimo Monturano, Gabriella Pravettoni

**Affiliations:** ^1^Applied Research Division for Cognitive and Psychological Science, European Institute of Oncology IRCCS, Milan, Italy; ^2^Department of Oncology and Hemato-Oncology, University of Milan, Milan, Italy; ^3^Patient Safety & Risk Management Service, European Institute of Oncology IRCCS, Milan, Italy; ^4^Department of Thoracic Surgery, European Institute of Oncology IRCCS, Milan, Italy

**Keywords:** lung cancer, quality of life, lobectomy, EORTC QLQ-C30, individual growth curve (IGS) models

## Abstract

**Objective:** Quality of Life (QoL) is an important predictor of patient's recovery and survival in lung cancer patients. The aim of the present study is to identify 1-year trends of lung cancer patients' QoL after robot-assisted or traditional lobectomy and investigate whether clinical (e.g., pre-surgery QoL, type of surgery, and perioperative complications) and sociodemographic variables (e.g., age) may predict these trends.

**Methods:** An Italian sample of 176 lung cancer patients undergoing lobectomy completed the European Organization for Research and Treatment of Cancer (EORTC) Quality of Life Questionnaire—Core 30 (QLQ-C30) at the pre-hospitalization (t0), 30 days (t1), 4 months (t2), 8 months (t3), and 12 months (t4) after surgery. Sociodemographic and clinical characteristics (age, gender, perioperative complications, and type of surgery) were also collected. The individual change over time of the 15 dimensions of the EORTC QLQ-C30 and the effects of pre-surgery scores of QoL dimensions, type of surgery, perioperative complications, and age on patients' QoL after surgery were studied with the individual growth curve (IGC) models.

**Results:** Patients had a good recovery after lobectomy: functioning subscales improved over time, while most of the symptoms became less severe over the care process. Perioperative complications, type of surgery, pre-surgery status, and age significantly affected these trends, thus becoming predictors of patients' QoL.

**Conclusion:** This study highlights different 1-year trends of lung cancer patients' QoL. The measurement of pre- and post-surgery QoL and its clinical and sociodemographic covariables would be necessary to better investigate patients' care process and implement personalized medicine in lung cancer hospital divisions.

## Introduction

Lung cancer is the most common cancer in both genders and the first cause of cancer death worldwide. Lung cancer trends are different among countries: Europe has lower trends than America (Siegel et al., 2016; Malvezzi et al., 2017; Bray et al., 2018). In Europe, the LucE Report (2016) stated that “more than 312,000 people were affected by lung cancer every year in the EU” (Lung Cancer Europe, 2016). In Italy, both the incidence and mortality rates are decreasing for men and increasing for women (Trama et al., 2017).

Primary malignant lung cancers are classified into two different categories: non-small-cell lung cancer (NSCLC) and small-cell lung cancer (SCLC); most lung cancer patients (about 80%) are diagnosed as NSCLC. Providing an overall 5-years survival rate of 55–77%, a resection surgical intervention is the recommended treatment for early-stage NSCLC (Polanski et al., 2016). Late diagnosis, comorbidities, and old age often impact on treatment possibilities, by reducing the therapeutic options and affecting patients' Quality of Life (QoL) (Ellis and Vandermeer, 2011; Iachina et al., 2015; Williams et al., 2016). Therefore, treatment efficacy, patient survival, and QoL are strictly related and mutually reinforcing. In this perspective, the QoL measurement is necessary to help the stakeholders having a more complete framework of patient's recovery and improving the decision-making process of the right treatment without being affected by cognitive biases (Mazzocco and Cherubini, 2010; Braun et al., 2011; Pravettoni et al., 2016).

The scientific literature shows indeed that QoL outcome, before and after surgery, is an important predictor of patient's recovery and survival in lung cancer patients (Herndon et al., 1999; Montazeri, 2009; Braun et al., 2011; Pierzynski et al., 2018). A systematic review (2009) (Montazeri, 2009) analyzing this association reported that most of the included articles indicate overall QoL, functional dysfunctions, and symptoms (e.g., pain, fatigue, and appetite loss)–adjusted for different sociodemographic and clinical characteristics–as prognostic factors of patients' survivorship. In fact, high survival rate is associated with better patients' well-being, higher motivation and engagement in doing physical activities, and greater pulmonary function (Rummans et al., 2006; Clark et al., 2008; Solberg Nes et al., 2012; Sterzi et al., 2013). Monitoring patients' QoL after surgery and identifying its predictors are therefore important to guarantee better survivorship: several studies showed that patients who underwent surgery often reported a worsening in QoL after treatment (Kenny et al., 2008). In particular, Yang et al. (2012) showed that 35% of long-term lung cancer survivors had a significant decline in overall QoL related to a worse level of fatigue, pain, dyspnea, appetite, and cough. Also, disturbed sleep and distress affect QoL over time (Lin et al., 2013). Another article (2013) analyzed demographic and clinical characteristics as predictors of QoL in lung cancer survivors and reported that younger participants showed more fatigue, dyspnea, and stress for financial problems. Patients with cancer-related comorbidities reported less severe dysphagia, nausea, and vomiting (Sterzi et al., 2013). According to the type of surgery, patients take 6–12 months to return to their preoperative QoL status (Dales et al., 1994; Handy et al., 2002). The video-assisted thoracoscopic surgery (VATS) implies a faster recovery and better QoL in NSCLC patients than the thoracotomy 1 year after surgery (Bendixen et al., 2016). Moreover, patients undergoing VATS were faster released from the hospital and reported less post-operative pain and complications than those who underwent traditional thoracotomy (Yang et al., 2017).

To our knowledge, only one previous research article studied the trajectories of lung cancer patients' QoL for 2 years after surgery. Kenny et al. (2008) showed that 65% of the recruited sample survived for 2 years after surgery and in that time QoL improved for patients with no recurrence, despite half of them continued to experience severe symptoms and functional limitations (Kenny et al., 2008). Nevertheless, the authors did not stratify for surgery type and did not study which sociodemographic or clinical characteristics may predict the QoL trend over time. For this reason, the aim of the present study is to identify 1-year trends of lung cancer patients' QoL after robot-assisted or traditional surgery and investigate whether clinical (e.g., pre-surgery QoL, type of surgery, and perioperative complications) and sociodemographic variables (e.g., age) may predict these trends.

## Materials and Methods

### Participants and Procedure

An Italian sample of 176 patients who underwent pulmonary lobectomy using the robotic-assisted approach or traditional open technique for lung cancer and participated in the Value Based Project^1^ were enrolled at the European Institute of Oncology in Milan between October 2015 and November 2017. Patients were included in the study if they: (1) were diagnosed with primary early-stage NSCLC (stage I and II), (2) were native Italian speakers, (3) were candidates for pulmonary lobectomy, and (4) had no neurological or psychopathological problems. Patients with cancer recurrences or with a previous thoracic surgical treatment were excluded from the study. All eligible patients were firstly asked to give written informed consent and then were asked to complete the European Organization for Research and Treatment of Cancer (EORTC) Quality of Life Questionnaire—Core 30 (QLQ-C30) questionnaire. They completed the EORTC QLQ-C30 at the pre-hospitalization (t0), 30 days (t1), 4 months (t2), 8 months (t3), and 12 months (t4) after lobectomy surgery.

Sociodemographic (i.e., age and gender) and clinical (i.e., perioperative complications: 0 = no perioperative complications, 1 = perioperative complications and type of surgery: 0 = traditional lobectomy, 1 = robot-assisted lobectomy) variables were also collected. Patients' sociodemographic and clinical characteristics are described in Table 1.

**Table 1:** Baseline characteristics of participants.

**Variables**	**Descriptive statistics**
Age, years [mean (SD)]	66.71 (7.68)
**Gender [*****N*** **(%)]**	
Female	70 (39.8%)
Male	106 (60.2%)
**Type of surgery [*****N*** **(%)]**	
Traditional lobectomy	117 (66.5%)
Robot-assisted lobectomy	59 (33.5%)
**Perioperative complications [*****N*** **(%)]**	
Yes	59 (33.5%)
No	117 (66.5%)
**Education [*****N*** **(%)]**	
<High school	70 (39.7%)
High school or equivalent	73 (41.5%)
>High school	22 (12.5%)
Unknown	11 (6.3%)

Most patients have completed data at every follow-up (55.7%). The 18.2% had missing data at one follow-up, 14.8% at two follow-ups, and 11.4% at three follow-ups. All data were collected and analyzed by a multidisciplinary team of the Value Based Project. The study was developed following the principles stated in the Declaration of Helsinki (59th WMA General Assembly, Seoul, 2008) and was approved by the European Institute of Oncology Ethical Committee at the European Institute of Oncology, Milan. Participation in the study was voluntary, and the patients could withdraw their consent at any time.

### Measures

The EORTC QLQ-C30 is the most commonly used tool for measuring QoL in lung cancer patients. Several studies reported good psychometric properties, demonstrating an excellent convergent and discriminant validity with the Functional Assessment of Cancer Therapy—General (FACT-G) questionnaire and good reliability for all domains (Cronbach's α higher than 0.70) (Jocham et al., 2009; Iravani et al., 2018; Marzorati et al., 2019b).

The EORTC QLQ-C30 consists of 30 self-reported questions assessing different aspects of patient functioning, global health status (GHS), and cancer-related symptoms. More specifically, it is composed of five multi-item functional scales (role, physical, cognitive, emotional, and social functioning), three multi-item symptom scales (fatigue, pain, nausea, and vomiting), individual items concerning common symptoms in cancer patients (dyspnea, insomnia, appetite loss, constipation, diarrhea, and financial difficulties), and two questions assessing overall QoL. All of the multi-item scales and single-item measures range in a score from 0 to 100. Specifically, a high score for a functional scale and overall QoL implicates a healthy level of functioning and GHS, whereas a high score for a symptom scale represents worse symptomatology (Aaronson et al., 1993).

### Statistical Analysis

Analyses were conducted using SPSS (Statistical Package for the Social Sciences), version 25. Individual growth curve (IGC) models with the SPSS MIXED procedure were performed to evaluate trends of post-operative QoL across time and to assess the influence of pre-surgery QoL, type of surgery, perioperative complications, and age on trends of QoL. IGC has several advantages in analyzing longitudinal data over traditional statistical methodologies, such as generalized linear models or analysis of variance. Specifically, IGC models allow one to validly analyze data that, as longitudinal data, violate the assumption of independence of observations. IGC models were performed by following the guidelines by Singer and Willett (2003) and Shek and Ma (2011) to validly assess longitudinal trends and interindividual differences in intraindividual changes over time. Specifically, the data were analyzed by using mixed effect models with maximum likelihood (ML) estimation. This method allowed to model individual change over time, determined the shape of the growth curves, and explored systematic differences in change by examining the effects of covariates (i.e., pre-surgery QoL, type of surgery, perioperative complications, and age) on QoL initial status and rate of growth. Each of the 15 EORTC-QLQ-C30 dimensions was analyzed separately in four consecutive steps.

In the first step, an unconditional mean model (i.e., Model 1) was tested. This is a one-way ANOVA model with a random effect with no predictors included. It served as a baseline model and assessed the intraclass correlation coefficient (ICC). ICC describes the amount of variance in each of the QoL dimensions that is attributed to differences between patients, and it evaluates the necessity of performing mixed model instead of traditional methods (e.g., ANOVA). Generally, an ICC of 0.25 or above indicates the necessity of performing longitudinal analysis with repeated measure mixed models.

The second step involved performing an unconditional linear growth model (Model 2) that explored linear individual variations in trends of QoL over time and served as a baseline model to assess whether the growth curve of QoL was linear or curvilinear. In the third step, an unconditional quadratic growth model (Model 3) was performed to assess whether the rate of change accelerated or decelerated across time following a parabola-shape.

The random effect for intercept was estimated in all the models; the random effect for linear change was estimated as well in Model 2 and Model 3. All these models were performed by fitting an unstructured (UN) covariance matrix for the random effects. Akaike Information Criterion (AIC) and −2log likelihood (−2LL) were considered to ascertain which of these three models were more appropriate to describe the change of each of the QoL dimensions over time. Specifically, the best fitting model was indicated by the lower values of AIC. Moreover, a statistically significant likelihood ratio test between a smaller model (i.e., lower number of estimated effects/parameters) vs. a more complex model indicated that the larger model provided a significant improvement in model fitting over the smaller one. Then, the best fitting model was subsequently retained and tested in the following steps. Specifically, in the last step, conditional models were performed to test whether pre-surgery QoL, age, type of surgery, and perioperative complications influenced initial QoL status at t1, linear growth rate, and quadratic change. Continuous variables (i.e., age and pre-surgery QoL) were grand mean centered, whereas perioperative complications and type of surgery were dummy coded (i.e., perioperative complications: 0 = no perioperative complications, 1 = perioperative complications and type of surgery: 0 = traditional lobectomy, 1 = robot-assisted lobectomy). Three different covariance structure models were performed to assess the error covariance structure: Model 4, conditional model with UN covariance structure; Model 5, conditional model with compound symmetry (CS) covariance structure; and Model 6, conditional model with first-order autoregressive (AR1) covariance structure. Once again, the best fitting model was identified by considering AIC and likelihood ratio test. In all the three models, the intercept and the slope were allowed to vary within individuals.

## Results

In the Appendix, Table A shows AIC, −2LL, and results of likelihood ratio tests for tested models for each of the 15 dimensions of QoL. ICC for Model 1 is reported as well. As reported, all ICCs were above 0.25 and ranged from 0.26 to 0.65. These results attested that it was necessary to perform longitudinal analysis with repeated measure mixed models for all the 15 considered dimensions of QoL.

### Global Health Status

**Figure 1:**
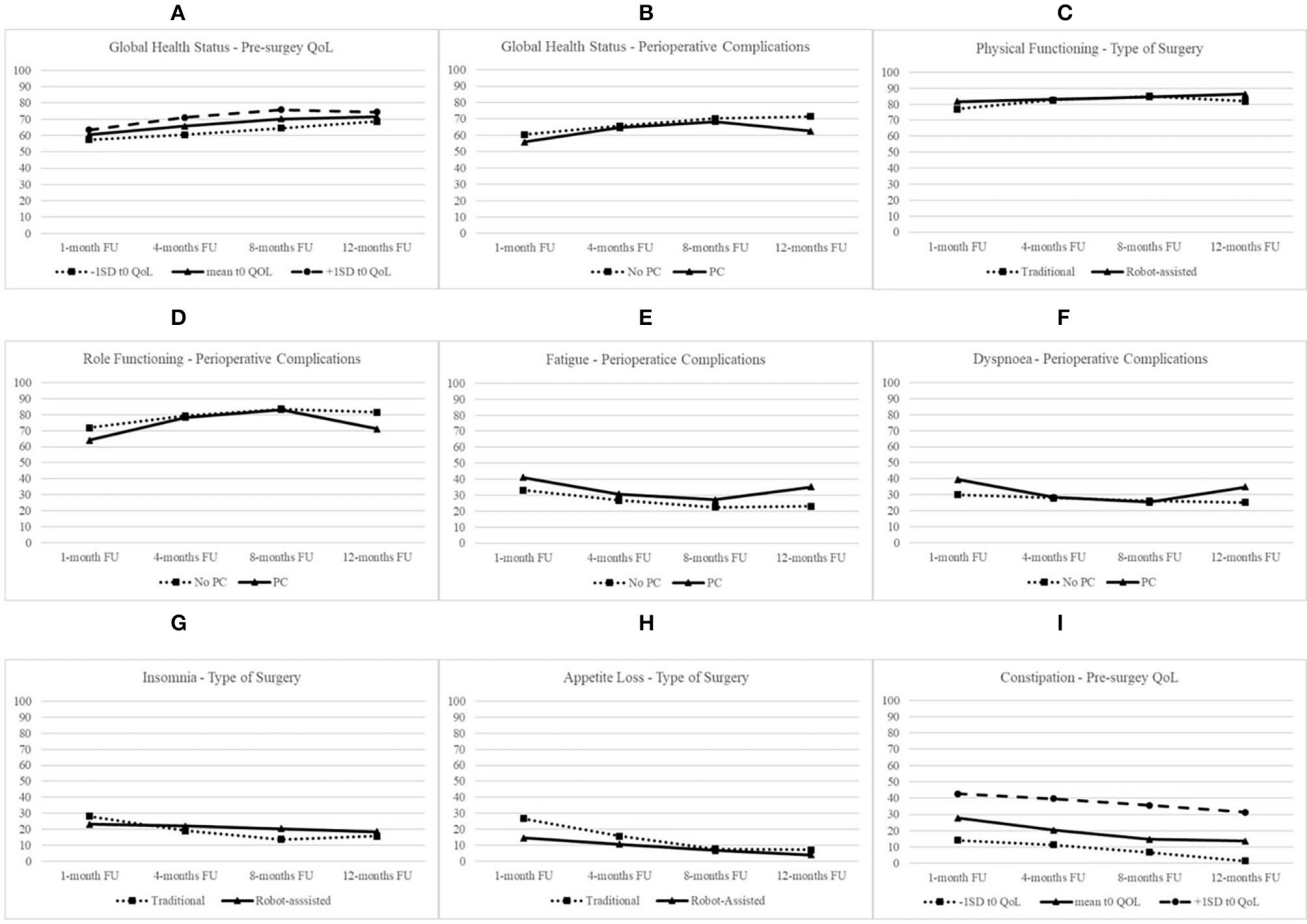
Longitudinal trends of QOL domains.

The best fit of Model 3 attested that there were significant between-subject variations in the initial level of GHS and linear and quadratic trajectories over time. Thus, this model was retained in subsequent analyses to test whether pre-surgery GHS, age, type of surgery, and perioperative complications influenced initial QoL status at t1, linear growth rate, and quadratic change and to compare the three error covariance structures. Model 6 with AR1 covariance structure showed the best fit. Table 2 reports of the final model with fixed effects for all the 15 dimensions of QoL. As shown, the initial level of GHS at t1 was 60.40 (S.E. = 2.05; *p* < 0.001), and it increased linearly over time (B = 2.07; S.E. = 0.71; *p* < 0.01). However, the rate of quadratic change was not significant (B = −0.10; S.E. = 0.06; n.s.). Patients with higher level of pre-surgery GHS showed higher subsequent level at t1 (B = 0.13; S.E. = 0.19; *p* < 0.05). As shown in Figure 1A, patients with a higher level of pre-surgery GHS reported a positive linear trend (B = 0.04; S.E. = 0.03; *p* < 0.05), indicating that their GHS increased more over time, and a negative quadratic rate of change indicating that their rate of growth decelerated more over time (B = −0.01; S.E. = 0.01; *p* < 0.05). On the contrary, the rate of quadratic change was negative for patients experiencing perioperative complications (B = −0.20; S.E. = 0.09; *p* < 0.05), indicating that their increasing effect gradually diminished more over time (Figure 1B).

**Table 2:** Fixed effects for all the 15 dimensions of QoL.

	**GHS**	**PF**	**RF**	**EF**	**CF**	**SF**	**FA**	**NV**
	**B (S.E.)**	**B (S.E.)**	**B (S.E.)**	**B (S.E.)**	**B (S.E.)**	**B (S.E.)**	**B (S.E.)**	**B (S.E.)**
Intercept	60.40 (2.05)***	77.05 (1.78)***	71.85 (2.54)***	76.18 (2.00)***	88.28 (1.51)***	82.49 (2.01)***	33.08 (2.22)***	8.93 (1.47)***
Time	2.07 (0.71)**	2.36 (0.53)***	3.10 (0.79)***	0.69 (0.27)*	−0.34 (0.19)^n.s.^	0.74 (0.22)**	−2.56 (0.73)**	−1.18 (0.41)**
TimeQ	−0.10 (0.06)^n.s.^	−0.17 (0.05)***	−0.20 (0.07)**	–	–	–	0.15 (0.07)*	0.07 (0.04)^n.s.^
T0	0.13 (0.07)*	0.52 (0.10)***	0.41 (0.10)***	0.48 (0.07)***	0.50 (0.06)***	0.35 (0.09)***	0.52 (0.08)***	0.12 (0.16)^n.s.^
TS at t0	4.69 (3.07)^n.s.^	4.66 (2.70)^n.s.^	7.04 (3.81)^n.s.^	3.80 (2.97)^n.s.^	3.37 (2.25)^n.s.^	5.40 (2.95)^n.s.^	−0.37 (3.36)^n.s.^	−4.23 (2.22)^n.s.^
PC	−4.64 (3.08)^n.s.^	−6.30 (2.73)*	−7.91 (2.87)*	−1.79 (2.98)^n.s.^	−1.00 (2.24)^n.s.^	−2.58 (3.00)^n.s.^	8.01 (3.35)*	0.11 (2.24)^n.s.^
Age	−0.13 (0.19)^n.s.^	−0.14 (0.17)^n.s^	0.32 (0.23)^n.s.^	−0.13 (0.18)^n.s.^	−0.16 (0.14)^n.s.^	0.28 (0.18)^n.s.^	0.17 (0.20)^n.s.^	−0.10 (0.13)^n.s.^
Time * t0 QoL	0.05 (0.02)*	0.01 (0.03)^n.s.^	−0.04 (0.03)^n.s.^	−0.02 (0.01)**	−0.01 (0.01)^n.s.^	−0.02 (0.01)^n.s.^	−0.05 (0.03)^n.s.^	−0.07 (0.04)^n.s.^
Time * TS	−1.17 (1.03)^n.s.^	−1.94 (0.80)*	−1.78 (1.18)^n.s.^	−0.49 (0.40)^n.s.^	0.11 (0.28)^n.s.^	−0.08 (0.33)^n.s.^	0.35 (1.09)^n.s.^	0.55 (0.62)^n.s.^
Time * PC	1.76 (1.05)^n.s.^	1.49 (0.83)^n.s.^	3.27 (1.21)**	−0.67 (0.41)^n.s.^	0.00 (0.29)^n.s.^	−0.40 (0.34)^n.s.^	−2.01 (1.10)^n.s.^	−0.33 (0.63)^n.s.^
Time * Age	−0.04 (0.07)^n.s.^	0.15 (0.05)^n.s.^	−0.09 (0.07)^n.s.^	0.02 (0.02)^n.s.^	0.01 (0.02)^n.s.^	−0.02 (0.02)^n.s.^	0.01 (0.07)^n.s.^	−0.02 (0.04)^n.s.^
TimeQ * t0 QoL	−0.01 (0.00)*	−0.00 (0.00)^n.s.^	0.00 (0.00)^n.s.^	–	–	–	0.00 (0.00)^n.s.^	0.01 (0.00)^n.s.^
TimeQ * TS	0.11 (0.09)^n.s.^	0.18 (0.07)*	0.19 (0.10)^n.s.^	–	–	–	−0.06 (0.10)^n.s.^	−0.04 (0.06)^n.s.^
TimeQ * PC	−0.20 (0.09)*	−0.14 (0.70)^n.s.^	−0.32 (0.11)**	–	–	–	0.22 (0.10)*	0.05 (0.06)^n.s.^
TimeQ * Age	0.01 (0.01)^n.s.^	0.00 (0.00)^n.s.^	0.01 (0.01)^n.s.^	–	–	–	−0.00 (0.00)^n.s.^	0.00 (0.00)^n.s.^
	**PA**	**DY**	**IN**	**AS**		**CO**	**DI**	**FD**
	**B (S.E.)**	**B (S.E.)**	**B (S.E.)**	**B (S.E.)**		**B (S.E.)**	**B (S.E.)**	**B (S.E.)**
Intercept	23.60 (2.39)***	29.95 (2.37)***	28.11 (2.76)***	26.63 (3.07)***		27.73 (3.15)***	6.73 (1.57)***	12.57 (2.03)***
Time	−1.58 (0.79)*	−0.77 (0.97)^n.s.^	−3.73 (0.93)***	−4.39 (0.88)***		−2.89 (0.98)**	−0.11 (0.24)^n.s.^	−0.03 (0.25)^n.s.^
TimeQ	0.06 (0.06)^n.s.^	0.03 (0.09)^n.s.^	0.24 (0.08)**	0.24 (0.08)**		0.15 (0.08)^n.s.^	–	–
T0	0.47 (0.11)***	0.42 (0.09)***	0.28 (0.08)***	.23 (0.13)^n.s.^		0.56 (0.09)***	0.37 (0.08)***	0.42 (0.06)***
TS at t0	−1.35 (3.61)^n.s.^	−4.04 (2.52)^n.s.^	−5.01 (4.11)^n.s.^	12.17 (4.56)**		−6.41 (4.69)^n.s.^	−2.36 (2.33)^n.s.^	−2.4 (2.98)^n.s.^
PC	4.84 (3.71)^n.s.^	9.41 (3.59)**	−0.87 (4.23)^n.s.^	−4.14 (4.65)^n.s.^		2.83 (4.70)^n.s.^	−2.88 (2.35)^n.s.^	0.69 (3.00)^n.s.^
Age	−0.34 (0.22)^n.s.^	−0.21 (0.22)^n.s.^	−0.13 (0.25)^n.s.^	0.26 (0.28)^n.s.^		0.33 (0.029)^n.s.^	−0.22 (0.14)^n.s.^	−0.40 (0.18)*
Time * t0 QoL	−0.06 (0.03)^n.s.^	−0.00 (0.04)^n.s.^	−0.01 (0.03)^n.s.^	−0.05 (0.04)^n.s.^		−0.08 (0.03)**	−0.02 (0.1)*	0.00 (0.01)^n.s.^
Time * TS	−1.45 (1.61)^n.s.^	0.22 (1.42)^n.s.^	3.37 (1.38)*	3.01 (1.31)*		1.00 (1.42)^n.s.^	0.14 (0.35)^n.s.^	−0.21 (0.37)^n.s.^
Time * PC	0.37 (1.22)^n.s.^	−4.06 (1.50)**	0.38 (1.42)^n.s.^	0.78 (1.34)^n.s.^		−0.53 (1.44)^n.s.^	0.35 (0.35)^n.s.^	0.88 (0.37)*
Time * Age	0.02 (0.07)^n.s.^	0.09 (0.09)^n.s.^	−0.10 (0.09)^n.s.^	−0.07 (0.08)^n.s.^		0.07 (0.09)^n.s.^	0.04 (0.02)^n.s.^	0.04 (003)^n.s.^
TimeQ * t0 QoL	0.01 (0.00)^n.s.^	−0.00 (0.00)^n.s.^	0.00 (0.00)^n.s.^	0.00 (0.00)^n.s.^		0.01 (0.00)**	–	–
TimeQ * TS	0.14 (0.10)^n.s.^	−0.08 (0.13)^n.s.^	−0.24 (0.12)*	−0.20 (0.11)^n.s.^		−0.1 (0.12)^n.s.^	–	–
TimeQ * PC	−0.03 (0.10)^n.s.^	0.37 (0.13)**	0.09 (0.12)^n.s.^	0.07 (0.12)^n.s.^		0.02 (0.12)^n.s.^	–	–
TimeQ * Age	0.00 (0.0)^n.s.^	−0.01 (0.01)^n.s.^	0.01 (0.01)^n.s.^	0.01 (0.01)^n.s.^		−0.01 (0.01)^n.s.^	–	–

GHS, global health status; PF, physical functioning; RF, role functioning; EF, emotional functioning; CF, cognitive functioning; SF, social functioning; FA, fatigue; NV, nausea and vomiting; PA, pain; DY, dyspnea; IN, insomnia; AS, appetite loss; CO, constipation; DI, diarrhea; FD, financial difficulties; Time, linear rate of growth; TimeQ, quadratic rate of growth; T0, pre-surgery subscales score; TS, type of surgery at t0; PC, perioperative complications; n.s., not significant. * = <0.05, ** = <0.01, and *** = <0.001.

### Physical Functioning

The best fit of Model 3 attested that there were significant between-subject variations in the initial level of physical functioning and linear and quadratic trajectories over time. Model 4 with UN covariance structure showed the best fit. As shown, the initial level of physical functioning at t1 was 77.05 (S.E. = 1.78; *p* < 0.001), increased linearly (B = 2.36; S.E. = 0.53; *p* < 0.001), and decelerated over time (B = −0.17; S.E. = 0.05; *p* < 0.001). Patients with higher pre-surgery physical functioning level (B = 0.52; S.E. = 0.10; *p* < 0.001) and without perioperative complications (B = −6.30; S.E. = 2.73; *p* < 0.01) showed higher subsequent level at t1. Type of surgery moderated rates of both linear and quadratic changes. Specifically, as shown in Figure 1C, patients undergoing robot-assisted surgery, compared with patients undergoing traditional surgery, reported a slower linear increase (B = −1.94; S.E. = 0.80; *p* < 0.05), but the positive quadratic effect (B = 0.18; S.E. = 0.07; *p* < 0.05) indicated that their rate of change decelerated less over time.

### Role Functioning

The best fit of Model 3 attested that there were significant between-subject variations in the initial level of role functioning and linear and quadratic trajectories over time. Model 4 with UN covariance structure showed the best fit. At t1, the level of role functioning was 71.85 (S.E. = 2.54; *p* < 0.001), increased linearly (B = 3.10; S.E. = 0.79; *p* < 0.001), and decelerated over time (B = −0.20; S.E. = 0.07; *p* < 0.01). Patients with higher pre-surgery role functioning level (B = 0.41; S.E. = 0.10; *p* < 0.001) and without perioperative complications (B = −7.91; S.E. = 3.87; *p* < 0.01) showed higher level of role functioning at t1. The experiencing of perioperative complications moderated rates of both linear and quadratic changes of role functioning. Specifically, as shown in Figure 1D, patients with perioperative complications, compared with patients without complications, reported a faster increase (B = 3.27; S.E. = 1.21; *p* < 0.001), but this increasing effect gradually diminished more (B = −0.32; S.E. = 0.11; *p* < 0.01) over time.

### Emotional Functioning

The best fit of Model 2 attested that there were significant between-subject variations in the initial level of emotional functioning and linear trajectories over time. Model 6 with AR1 covariance structure showed the best fit. Emotional functioning at t1 was 76.18 (S.E. = 2.00; *p* < 0.001) and increased linearly over time (B = 0.69; S.E. = 0.27; *p* < 0.01). Patients with higher pre-surgery emotional functioning level (B = 0.48; S.E. = 0.10; *p* < 0.001) showed higher level at t1. Moreover, the level of pre-surgery emotional functioning moderated rates of linear change of emotional functioning. Specifically, patients with higher pre-surgery level reported a slower increase of emotional functioning over time (B = −0.02; S.E. = 0.01; *p* < 0.01).

### Cognitive Functioning

The best fit of Model 2 attested that there were significant between-subject variations in the initial level of cognitive functioning and linear trajectories over time. Model 4 with UN covariance structure showed the best fit. At t1, cognitive functioning was 88.28 (S.E. = 1.51; *p* < 0.001) and did not linearly increase over time (B = −0.34; S.E. = 0.19; n.s.). Patients with higher pre-surgery cognitive functioning level showed higher level at t1 (B = 0.50; S.E. = 0.06; *p* < 0.001). Any of the sociodemographic or clinical variables influenced the linear rate of change over time.

### Social Functioning

The best fit of Model 2 attested that there were significant between-subject variations in the initial level of social functioning and linear trajectories over time. Model 5 with CS covariance structure showed the best fit. Social functioning at t1 was 82.49 (S.E. = 2.01; *p* < 0.001) and increased linearly over time (B = 0.74; S.E. = 0.22; *p* < 0.01). Patients with higher pre-surgery social functioning level showed higher level of social functioning at t1 (B = 0.35; S.E. = 0.09; *p* < 0.001). Any of the sociodemographic or clinical variables influenced the linear rate of change of social functioning over time.

### Fatigue

The best fit of Model 3 attested that there were significant between-subject variations in the initial level of fatigue and linear and quadratic trajectories over time. Model 5 with CS covariance structure showed the best fit. At t1, the level of fatigue was 33.08 (S.E. = 2.22; *p* < 0.001) and decreased linearly (B = −2.56; S.E. = 0.73; *p* < 0.01) but decelerated over time (B = 0.15; S.E. = 0.07; *p* < 0.05). Patients with higher level of pre-surgery fatigue (B = 0.52; S.E. = 0.08; *p* < 0.001) and with perioperative complications (B = 8.01; S.E. = 3.35; *p* < 0.05) showed higher level of fatigue at t1. The experiencing of perioperative complications moderated the quadratic rate of growth of fatigue over time. Specifically, as shown in Figure 1E, patients with perioperative complications, compared with patients without complications, reported a higher deceleration of decreasing rate of fatigue over time (B = 0.22; S.E. = 0.10; *p* < 0.05).

### Nausea and Vomiting

The best fit of Model 3 attested that there were significant between-subject variations in the initial level of nausea and vomiting and linear and quadratic trajectories over time. Model 4 with UN covariance structure showed the best fit. After the inclusion of predictors, the level of nausea and vomiting at t1 was 8.938 (S.E. = 1.47; *p* < 0.001) and decreased linearly over time (B = −1.18; S.E. = 0.41; *p* < 0.01). Any of the sociodemographic or clinical variables influenced the initial status of nausea and vomiting, neither linear nor quadratic rate of change of fatigue over time.

### Pain

The best fit of Model 3 attested that there were significant between-subject variations in the initial level of pain and linear and quadratic trajectories over time. Model 4 with UN covariance structure showed the best fit. Pain at t1 was 23.60 (S.E. = 2.39; *p* < 0.001) and decreased linearly over time (B = −1.58; S.E. = 0.79; *p* < 0.05). Patients with higher pre-surgery pain showed a higher level of this QoL dimension at t1 (B = 0.47; S.E. = 0.11; *p* < 0.001).

### Dyspnea

The best fit of Model 3 attested that there were significant between-subject variations in the initial level of dyspnea and linear and quadratic trajectories over time. Model 5 with CS covariance structure showed the best fit. At t1, the level of dyspnea was 29.95 (S.E. = 2.37; *p* < 0.001), but it did not increase linearly (B = −0.77; S.E. = 2.37; n.s.) neither accelerated over time (B = −0.77; S.E. = 0.98; n.s.). Patients with higher level of pre-surgery dyspnea (B = 0.42; S.E. = 0.09; *p* < 0.001) and with perioperative complications (B = 9.41; S.E. = 3.59; *p* < 0.01) showed higher level of this QoL dimension at t1. The experiencing of perioperative complications moderated rates of both linear and quadratic changes of dyspnea. Specifically, as shown in Figure 1F, patients with perioperative complications, compared with patients without complications, reported a steeper decrease (B = −5.06; S.E. = 1.47; *p* < 0.01) but a faster deceleration of decreasing effect over time (B = 0.37; S.E. = 0.13; *p* < 0.01).

### Insomnia

The best fit of Model 3 attested that there were significant between-subject variations in the initial level of insomnia and linear and quadratic trajectories over time. Model 6 with AR1 covariance structure showed the best fit. As shown, the initial level of insomnia at t1 was 28.11 (S.E. = 2.76; *p* < 0.001), decreased linearly (B = −3.73; S.E. = 0.93; *p* < 0.001), and decelerated over time (B = 0.24; S.E. = 0.08; *p* < 0.01). Patients with higher level of pre-surgery insomnia showed higher level at t1 (B = 0.28; S.E. = 0.08; *p* < 0.001). Type of surgery moderated rates of both linear and quadratic changes. Specifically, as shown in Figure 1G, patients undergoing robot-assisted surgery, compared with patients undergoing traditional surgery, reported a slower linear change (B = 3.37; S.E. = 1.38; *p* < 0.05) but a slower deceleration of decreasing effect over time (B = −0.24; S.E. = 0.12; *p* < 0.05).

### Appetite Loss

The best fit of Model 3 attested that there were significant between-subject variations in the initial level of appetite loss and linear and quadratic trajectories over time. Model 4 with UN covariance structure showed the best fit. Appetite loss at t1 was 26.63 (S.E. = 3.07; *p* < 0.001), decreased linearly (B = −4.39; S.E. = 0.88; *p* < 0.001), and decelerated over time (B = 0.24; S.E. = 0.08; *p* < 0.01). Patients undergoing robot-assisted surgery showed lower level of appetite loss at t1 (B = 12.17; S.E. = 4.56; *p* < 0.01). Moreover, type of surgery moderated rates of linear change of appetite loss over time. Specifically, as shown in Figure 1H, patients undergoing robot-assisted surgery, compared with patients undergoing traditional surgery, reported a slower linear decrease over time (B = 3.01; S.E. = 1.31; *p* < 0.05).

### Constipation

The best fit of Model 3 attested that there were significant between-subject variations in the initial level of constipation and linear and quadratic trajectories over time. Model 4 with UN covariance structure showed the best fit. At t1, the initial level of constipation was 27.73 (S.E. = 3.15; *p* < 0.001), and it decreased linearly over time (B = −2.89; S.E. = 0.97; *p* < 0.01). Patients with higher level of pre-surgery constipation showed higher subsequent level at t1 (B = 0.56; S.E. = 0.09; *p* < 0.001). As shown in Figure 1I, the decreasing effect was faster for patients with higher level of pre-surgery constipation (B = −0.08; S.E. = 0.03; *p* < 0.01), but it showed less acceleration of decreasing effect over time (B = 0.01; S.E. = 0.00; *p* < 0.01).

### Diarrhea

The best fit of Model 2 attested that there were significant between-subject variations in the initial level of diarrhea and linear trajectories over time. Model 4 with UN covariance structure showed the best fit. Diarrhea t1 was 6.73 (S.E. = 1.57; *p* < 0.001), but after the inclusion of sociodemographic and clinical predictors, it did not linearly change over time (B = −0.11; S.E. = 0.24; n.s.). Patients with higher level of pre-surgery diarrhea showed higher level at t1 (B = 0.37; S.E. = 0.08; *p* < 0.001) and reported a faster decrease over time (B = −0.02; S.E. = 0.01; *p* < 0.5).

### Financial Difficulties

The best fit of Model 2 attested that there were significant between-subject variations in the initial level of financial difficulties and linear trajectories over time. Model 4 with UN covariance structure showed the best fit. At t1, financial difficulties score was 12.57 (S.E. = 2.03; *p* < 0.001), but after the inclusion of sociodemographic and clinical predictors, it did not linearly change over time (B = −0.04; S.E. = 0.25; n.s.). Patients with higher level of pre-surgery financial difficulties (B = 0.42; S.E. = 0.06; *p* < 0.001) and younger patients (B = −0.40; S.E. = 0.18; *p* < 0.05) reported higher level at t1. Moreover, patients with perioperative complications reported a faster increase of financial difficulties over time than patients without this kind of difficulties (B = 0.88; S.E. = 0.37; *p* < 0.05).

## Discussion

The present study identified 1-year trends of patients' QoL after pulmonary lobectomy for NSCLC and investigated whether clinical and sociodemographic variables may predict these trends. The individual change over time of the 15 dimensions of the EORTC QLQ-C30 and the effects of pre-surgery scores of QoL dimensions, type of surgery, perioperative complications, and age on patients' QoL after surgery were studied with the IGC models.

According to other previous studies (Pompili, 2015), our results showed that patients had a good recovery after lobectomy. This is attested by an overall decrease in symptoms and an increase of health and functioning over time. However, levels of QoL at pre-surgery, type of surgery, perioperative complications, and patient's age generally affected the post-surgery initial status of QoL as well as its linear and quadratic trends over time. This overall recovery in QoL is quite clear by looking at the results concerning the GHS. Specifically, patients' health increased linearly over time. Pre-surgery GHS significantly affected this trend after lobectomy: lung cancer patients with high levels of pre-surgery GHS had better score 30 days after surgery and better 1-year recovery, even if their beneficial trend tended to slow down over time. Also, patients experiencing perioperative complications, compared with people with no complications, reported a greater deceleration of the recovery rate over time, suggesting that these kinds of patients are likely to experience a late relapse of global health.

Referring to the European reference values for the QoL questionnaire EORTC QLQ-C30 (2008) (Scott et al., 2008), 1 month after surgery, patients globally reported high physical, role, emotional, cognitive, and social functioning score rates, varying from 71.85 (role function) to 88.28 (cognitive function). Except for cognitive functioning (presenting high levels at all time), all the other functioning subscales linearly increased over time, showing a fast and good recovery after surgery. Only the recovery trend of physical and role functioning significantly decelerated over time: the linear improvement of both functions was faster in the first months after surgery and tended to become slower as time went on. Emotional, cognitive, and social subscales constantly increased over time, indicating that patients are likely to have a good psychosocial recovery after surgery. These findings are in line with a recent systematic review (2015) on QoL after lung cancer resection, showing that physical functioning is the most affected dimension in patients with NSCLC, but all the EORTC QLQ-C30 subscales generally recover in 3–12 months after surgery (Pompili, 2015). The analysis of patients' trends and time of functioning recovery may promote the identification of specific intermediate and long-term effects over the care process and add valuable information in understanding QoL trajectories (Balduyck et al., 2007).

Pre-surgery levels significantly impacted all functioning subscales 1 month after surgery: patients with high levels of physical, role, emotional, cognitive, and social functions before surgery showed higher levels even 1 month after surgery. Moreover, patients with higher pre-surgery emotional functioning had a slower improvement of QoL over time. Among the other aspects that may affect patients' functions, the type of surgery significantly impacted only the linear and quadratic trends of physical functioning over time. Specifically, compared with patients undergoing robot-assisted surgery, people undergoing traditional surgery displayed a faster linear improvement in physical functioning after lobectomy, but this recovery remained less stable over time for patients undergoing traditional surgery. Balduyck et al. (2007), analyzing patients undergoing traditional or robotic-assisted surgery, demonstrated that patients undergoing traditional lobectomy had the worst effects on physical functioning and pain over 1 year. Our results attested that patients' functioning may be affected also by perioperative complications as well. Specifically, lung cancer patients with perioperative complications had lower scores in physical and role functions 30 days after surgery but a faster recovery from role functioning impairment. However, the speed of recovery from emotional problems tended to slow down more at a later time (or become even worse) for patients experiencing complications.

The symptom subscale trends were also investigated. Patients' reported symptoms 1 month after surgery were in line with the reference score values (Scott et al., 2008) of lung cancer. Only dyspnea and constipation symptoms were lower than their reference score means: 30 days after surgery, patients reported dyspnea of 29.95 and constipation of 27.73, whereas the average means are 42.7 and 15.0, respectively. Since higher rates indicate worse symptoms, lung cancer patients undergoing lobectomy in our study had fewer problems of dyspnea and more constipation than expected. This could be explained by the continuous use of post-operative pain killers even after 30 days conditioning a better pulmonary function due to less pain but increasing constipation. The 1-year trend linearly decreased for fatigue, pain, insomnia, appetite loss, and constipation, suggesting a recovery from symptoms over time. However, the speed of recovery gradually slowed down for fatigue and appetite loss as the time from surgery went on, prolonging patients' tiredness and inappetence.

Pre-surgery levels significantly impacted fatigue, pain, dyspnea, insomnia, constipation, diarrhea, and financial difficulties 1 month after surgery: patients with high levels of these symptoms before surgery showed higher problems even 30 days after surgery. Moreover, patients with higher pre-surgery levels of constipation and diarrhea had a faster decrease in these symptoms over time, but the first one showed a slower recovery in the last months of the 1-year trend. The type of surgery significantly impacted insomnia and appetite loss rates 1 month after surgery and over time: patients undergoing robot-assisted surgery had low scores 30 days after surgery but a slower improvement after lobectomy in these symptoms than patients undergoing traditional surgery. This slow improvement is probably due to the favorable initial condition: patients undergoing robot-assisted surgery had fewer symptoms 1 month after surgery, and they may not further improve over time since they already had high scores 30 days after surgery. However, the significant negative quadratic change of insomnia showed that it remained more stable over time for patients undergoing robot-assisted lobectomy, suggesting that patients undergoing traditional surgery were more likely to experience a worsening of sleep problems at a later time. Perioperative complications significantly affected the 1 month scores of dyspnea and fatigue: lung cancer patients with perioperative complications had higher levels of dyspnea and fatigue 30 days after surgery. Moreover, complications affected the recovery of dyspnea and financial difficulties over time: patients with no complications had a faster increase of dyspnea symptom and a slower increase in financial difficulties and spent less money. Finally, the quadratic change of dyspnea and fatigue was impacted by perioperative complications, showing a greater slowdown of the recovery for patients with complications. Age significantly impacted only on financial difficulties: younger patients had greater financial problems 30 days after surgery than older people.

The obtained results identify different 1-year trends of lung cancer patients' QoL after lobectomy. All sub-dimensions had a specific recovery: functioning subscales improved over time, whereas most of the symptoms became less severe over the care process. Perioperative complications, type of surgery, pre-surgery status, and age significantly affected these trends, thus becoming predictors of patients' QoL. In fact, in this paper, it was often demonstrated that pre-surgery QoL rates often predicted post-surgery status and trends, whereas the type of surgery, age, and perioperative complications often affect patients' well-being and recovery. Therefore, the measurement of pre- and post-surgery QoL and its clinical and sociodemographic covariables would be necessary to better investigate patients' care process and implement personalized medicine in lung cancer hospital divisions. A patient-centered approach would be important to develop preventive programs, analyze both psychological and medical outcomes that could affect patient's recovery, and improve patient empowerment (Marzorati et al., 2018; Bailo et al., 2019).

Current results may be considered in light of some main limitations. Specifically, because of sample size, it was not possible to identify different typologies of patients following different longitudinal trajectories of QoL: 176 patients with lung cancer were not enough to distinguish different trends of recovery. This study shows the average 1-year trend, but it did not identify different classes of patients with different recoveries after surgery. Future studies should be conducted on a larger sample in order to perform other statistical analyses with a typological approach that can better describe patients' recovery. It would be also important to collect data on the effects of other psychological aspects that may significantly impact the trend of patients' QoL. For example, illness perception, resilience, coping, and self-efficacy are only some of the important aspects that should be measured over the care cycle and may modify patients' recovery after surgery (Greco et al., 2015; Hu et al., 2018; Oliveri et al., 2019). Interpersonal variables, such as patient–physician communication and trust, are other factors that may affect patients' care process (Kenny et al., 2010; Petrocchi et al., 2019). Future studies are needed to better identify covariables that may impact on lung cancer patients' QoL and identify different trajectories of patients' recovery (Marzorati et al., 2019a). Moreover, measured outcomes were collected up to only 1 year after surgery: it would be important to extend the follow-ups, in order to better analyze patient's recovery of functions, which mostly lasts more than 1 year after treatments. Lastly, since QoL is strictly associated with survivorship rates, it would be useful to conduct another project studying which QoL sub-dimensions may interact or affect patients' survivorship (Montazeri, 2009).

Despite these limitations, this study not only identifies trends of lung cancer patients' QoL after robot-assisted or traditional surgery but also provides new evidences on patients' clinical and sociodemographic characteristics that may predict and better describe patients' recovery over the care process. These evidences may be important elements to be discussed during medical consultations: physicians may adopt this information to help patients make informed decisions, complying with their expectations and preferences about long-term outcomes (Pompili, 2015).

## Conclusion

Lung cancer patients who underwent robot-assisted or traditional lobectomy and were followed up for 1-year showed the individual change in the 15 dimensions of the EORTC QLQ-C30. Pre-surgery scores of QoL dimensions, type of surgery, perioperative complications, and age significantly affected the post-surgery initial status of QoL as well as its linear and quadratic trends over time. Pre- and post-surgery QoL and its clinical and sociodemographic covariables should be always measured to better investigate patients' care process and implement personalized programs.

## Data Availability Statement

The datasets generated for this study are available on request to the corresponding author.

## Ethics Statement

The studies involving human participants were reviewed and approved by Ethical Committee of the European Institute of Oncology, Milan. The patients/participants provided their written informed consent to participate in this study.

## Author Contributions

All authors listed have made a substantial, direct and intellectual contribution to the work, and approved it for publication.

## Conflict of Interest

The authors declare that the research was conducted in the absence of any commercial or financial relationships that could be construed as a potential conflict of interest.

## Footnotes

^1^The Value Based Project is a multidisciplinary project where medical, economical, and psychological outcomes are collected through standardized questionnaires at different time points consistently with patient's recovery and follow-ups. The aim of this project is to create predictive models of care pathways of the major cancer diagnosis.
