# Supplementary material for: One-Year Quality of Life Trends in Early-Stage Lung Cancer Patients After Lobectomy
Source: Front Psychol. 2020 Dec 10;11:534428. doi: 10.3389/fpsyg.2020.534428 (PMC7758417; doi:10.3389/fpsyg.2020.534428)
Supplement: Supplementary file 1 [file Table_1.DOCX]

*Table A. ICC, AIC, -2LL and results of likelihood ratio tests for tested models (underlined the best fitting model among M1, M2 and M3; in italics the best fitting model among M4, M5 and M6).*

| QoL | Model | ICC | AIC | -2LL | #EP | Comparison | Δ-2LL | Δdf | p |
| --- | --- | --- | --- | --- | --- | --- | --- | --- | --- |
| GHS | M1 | .50 | 4461.49 | 4455.49 | 3 | - | - | - | - |
|  | M2 | - | 4407.33 | 4395.33 | 6 | M1-M2 | 60.16 | 3 | *** |
|  | M3 | - | 4399.45 | 4385.45 | 7 | M2-M3 | 9.88 | 1 | ** |
|  | M4 | - | 4282.58 | 4232.58 | 25 | - | - | - | - |
|  | M5 | - | 4303.05 | 4269.05 | 17 | M4-M5 | 36.47 | 8 | *** |
|  | *M6* | *-* | *4276.52* | *4242.52* | *17* | *M4-M6* | *9.94* | *8* | *n.s.* |
| PF | M1 | .60 | 4464.18 | 4458.18 | 3 | - | - | - | - |
|  | M2 | - | 4445.56 | 4433.56 | 6 | M1-M2 | 24.63 | 3 | *** |
|  | M3 | - | 4420.28 | 4406.28 | 7 | M2-M3 | 27.28 | 1 | *** |
|  | *M4* | *-* | *4225.07* | *4175.07* | *25* | *-* | *-* | *-* | *-* |
|  | M5 | - | 4242.02 | 4208.02 | 17 | M4-M5 | 32.95 | 8 | *** |
|  | M6 | - | 4253.14 | 4219.14 | 17 | M4-M6 | 44.07 | 8 | *** |
| RF | M1 | .54 | 4867.23 | 4861.23 | 3 | - | - | - | - |
|  | M2 | - | 4831.91 | 4819.91 | 6 | M1-M2 | 41.33 | 3 | *** |
|  | M3 | - | 4810.66 | 4796.66 | 7 | M2-M3 | 23.25 | 1 | *** |
|  | *M4* | *-* | *4604.95* | *4554.95* | *25* | *-* | *-* | *-* | *-* |
|  | M5 | - | 4614.12 | 4580.12 | 17 | M4-M5 | 25.17 | 8 | ** |
|  | M6 | - | 4621.59 | 4587.59 | 17 | M4-M6 | 32.64 | 8 | *** |
| EF | M1 | .53 | 4422.01 | 4416.01 | 3 | - | - | - | - |
|  | M2 | - | 4386.90 | 4374.90 | 6 | M1-M2 | 41.12 | 3 | *** |
|  | M3 | - | 4387.88 | 4373.88 | 7 | M2-M3 | 1.02 | 1 | n.s. |
|  | M4 | - | 4138.71 | 4098.71 | 20 | - | - | - | - |
|  | M5 | - | 4155.16 | 4131.16 | 12 | M4-M5 | 32.45 | 8 | *** |
|  | *M6* | *-* | *4135.03* | *4111.03* | *12* | *M4-M6* | *12.32* | *8* | *n.s.* |
| CF | M1 | .65 | 4274.46 | 4268.46 | 3 | - | - | - | - |
|  | M2 | - | 4250.06 | 4238.06 | 6 | M1-M2 | 30.40 | 3 | *** |
|  | M3 | - | 4252.01 | 4238.01 | 7 | M2-M3 | 0.05 | 1 | n.s. |
|  | *M4* | *-* | *4074.94* | *4034.94* | *20* | *-* | *-* | *-* | *-* |
|  | M5 | - | 4091.37 | 4067.37 | 12 | M4-M5 | 32.43 | 8 | *** |
|  | M6 | - | 4090.10 | 4066.10 | 12 | M4-M6 | 31.16 | 8 | *** |
| SF | M1 | .52 | 4495.40 | 4489.40 | 3 | - | - | - | - |
|  | M2 | - | 4482.41 | 4470.41 | 6 | M1-M2 | 18.99 | 3 | *** |
|  | M3 | - | 4481.66 | 4467.66 | 7 | M2-M3 | 2.74 | 1 | n.s. |
|  | M4 | - | 4376.62 | 4336.62 | 20 | - | - | - | - |
|  | *M5* | *-* | *4376.09* | *4352.09* | *12* | *M4-M5* | *15.48* | *8* | *n.s.* |
|  | M6 | - | 4386.86 | 4362.86 | 12 | M4-M6 | 26.24 | 8 | ** |
| FA | M1 | .61 | 4533.00 | 4527.00 | 3 | - | - | - | - |
|  | M2 | - | 4500.74 | 4488.74 | 6 | M1-M2 | 38.26 | 3 | *** |
|  | M3 | - | 4482.64 | 4468.64 | 7 | M2-M3 | 20.10 | 1 | *** |
|  | M4 | - | 4268.79 | 4218.79 | 25 | - | - | - | - |
|  | *M5* | *-* | *4259.61* | *4225.61* | *17* | *M4-M5* | *6.82* | *8* | *n.s.* |
|  | M6 | - | 4287.78 | 4253.78 | 17 | M4-M6 | 34.99 | 8 | ** |
| NV | M1 | .43 | 4271.99 | 4265.99 | 3 | - | - | - | - |
|  | M2 | - | 4235.73 | 4223.73 | 6 | M1-M2 | 42.27 | 3 | *** |
|  | M3 | - | 4233.48 | 4219.48 | 7 | M2-M3 | 4.25 | 1 | * |
|  | *M4* | *-* | *4065.67* | *4015.67* | *25* | *-* | *-* | *-* | *-* |
|  | M5 | - | 4134.39 | 4100.40 | 17 | M4-M5 | 84.72 | 8 | *** |
|  | M6 | - | 4122.28 | 4088.28 | 17 | M4-M6 | 72.60 | 8 | *** |

| QoL | Model | ICC | AIC | -2LL | #EP | Comparison | Δ-2LL | Δdf | p |
| --- | --- | --- | --- | --- | --- | --- | --- | --- | --- |
| PA | M1 | .54 | 4570.56 | 4564.56 | 3 | - | - | - | - |
|  | M2 | - | 4530.85 | 4518.85 | 6 | M1-M2 | 45.71 | 3 | *** |
|  | M3 | - | 4523.99 | 4509.99 | 7 | M2-M3 | 8.86 | 1 | ** |
|  | *M4* | *-* | *4159.21* | *4109.21* | *25* | *-* | *-* | *-* | *-* |
|  | M5 | - | 4186.29 | 4152.29 | 17 | M4-M5 | 43.07 | 8 | *** |
|  | M6 | - | 4193.91 | 4159.91 | 17 | M4-M6 | 50.70 | 8 | *** |
| DY | M1 | .38 | 4987.10 | 4981.10 | 3 | - | - | - | - |
|  | M2 | - | 4980.15 | 4968.15 | 6 | M1-M2 | 12.95 | 3 | ** |
|  | M3 | - | 4976.78 | 4962.78 | 7 | M2-M3 | 5.37 | 1 | * |
|  | M4 | - | 4789.35 | 4739.35 | 25 | - | - | - | - |
|  | *M5* | *-* | *4780.63* | *4746.63* | *17* | *M4-M5* | *7.28* | *8* | *n.s.* |
|  | M6 | - | 4792.89 | 4758.89 | 17 | M4-M6 | 19.54 | 8 | ** |
| IN | M1 | .55 | 5041.78 | 5035.78 | 3 | - | - | - | - |
|  | M2 | - | 5024.71 | 5012.71 | 6 | M1-M2 | 23.07 | 3 | *** |
|  | M3 | - | 5013.63 | 4999.63 | 7 | M2-M3 | 13.08 | 1 | *** |
|  | M4 | - | 4832.47 | 4782.47 | 25 | - | - | - | - |
|  | M5 | - | 4841.44 | 4807.44 | 17 | M4-M5 | 24.97 | 8 | ** |
|  | *M6* | *-* | *4831.96* | *4797.96* | *17* | *M4-M6* | *15.48* | *8* | *n.s.* |
| AS | M1 | .46 | 4996.77 | 4990.77 | 3 | - | - | - | - |
|  | M2 | - | 4934.42 | 4922.42 | 6 | M1-M2 | 68.35 | 3 | *** |
|  | M3 | - | 4923.87 | 4909.87 | 7 | M2-M3 | 12.55 | 1 | *** |
|  | *M4* | *-* | *4727.52* | *4677.52* | *25* | *-* | *-* | *-* | *-* |
|  | M5 | - | 4764.70 | 4730.70 | 17 | M4-M5 | 53.17 | 8 | *** |
|  | M6 | - | 4746.70 | 4712.70 | 17 | M4-M6 | 35.18 | 8 | *** |
| CO | M1 | .48 | 4848.39 | 4842.39 | 3 | - | - | - | - |
|  | M2 | - | 4799.54 | 4787.54 | 6 | M1-M2 | 54.85 | 3 | *** |
|  | M3 | - | 4793.15 | 4779.15 | 7 | M2-M3 | 8.39 | 1 | *** |
|  | *M4* | *-* | *4675.50* | *4625.50* | *25* | *-* | *-* | *-* | *-* |
|  | M5 | - | 4716.71 | 4682.71 | 17 | M4-M5 | 57.21 | 8 | *** |
|  | M6 | - | 4702.85 | 4668.85 | 17 | M4-M6 | 43.35 | 8 | *** |
| DI | M1 | .26 | 4333.62 | 4327.62 | 3 | - | - | - | - |
|  | M2 | - | 4313.64 | 4301.64 | 6 | M1-M2 | 25.97 | 3 | *** |
|  | M3 | - | 4315.12 | 4301.12 | 7 | M2-M3 | 0.52 | 1 | n.s. |
|  | *M4* | *-* | *4260.42* | *4220.42* | *20* | *-* | *-* | *-* | *-* |
|  | M5 | - | 4273.76 | 4249.76 | 12 | M4-M5 | 22.17 | 8 | *** |
|  | M6 | - | 4261.01 | 4237.01 | 12 | M4-M6 | 9.42 | 8 | * |
| FD | M1 | .56 | 4614.05 | 4608.05 | 3 | - | - | - | - |
|  | M2 | - | 4610.34 | 4598.34 | 6 | M1-M2 | 9.71 | 3 | * |
|  | M3 | - | 4612.34 | 4598.34 | 7 | M2-M3 | 0.00 | 1 | n.s. |
|  | *M4* | *-* | *4472.48* | *4432.48* | *20* | *-* | *-* | *-* | *-* |
|  | M5 | - | 4488.43 | 4464.43 | 12 | M4-M5 | 31.95 | 8 | *** |
|  | M6 | - | 4514.41 | 4490.41 | 12 | M4-M6 | 57.93 | 8 | *** |

*Note: GHS = Global health status; PF = Physical functioning; RL = Role functioning; EF = Emotional functioning; CF = Cognitive functioning; SF = Social functioning; FA = Fatigue; NV = Nausea and vomiting; PA = Pain; DY = Dyspnoea; IN = Insomnia; AS = Appetite loss; CO =Constipation; DI = Diarrhoea; FD = Financial difficulties; ICC = Intraclass correlation; AIC = Akaike Information Criterion; -2LL = -2log likelihood; #EP = Number of estimated parameters; Δ-2LL = Differences in -2log likelihood between compared models; Δdf = Differences in number of estimated parameters/degrees of freedom*
